# Contrasting Reproductive and Early Life‐History Strategies in Recently Diverged Octocoral Species (*Paramuricea* spp.)

**DOI:** 10.1002/ece3.73264

**Published:** 2026-03-30

**Authors:** Christina Egger, Aschwin H. Engelen, Roland R. Melzer, Marcellina Rola, Catarina Melo, Chiara Favaretto, Manuela Quiroga‐Pérez, Sheena Suet‐Wah Chung, Lorenzo Bramanti, Ester A. Serrão, Márcio A. G. Coelho

**Affiliations:** ^1^ Centro de Ciências Do Mar Do Algarve (CCMAR/CIMAR LA), Campus de Gambelas Universidade Do Algarve Faro Portugal; ^2^ SNSB ‐ Bavarian State Collection of Zoology Munich Germany; ^3^ Caribbean Research and Management of Biodiversity Foundation Willemstad Curaçao; ^4^ LMU Munich GeoBio‐Center Munich Germany; ^5^ Department of Evolution and Biodiversity Bergische Universitat Wuppertal Wuppertal Germany; ^6^ Campus de Gambelas Universidade Do Algarve Faro Portugal; ^7^ Department Biology University of Padua Padua Italy; ^8^ Zoological Department III Natural History Museum Vienna Vienna Austria; ^9^ Department of Functional and Evolutionary Ecology University of Vienna Vienna Austria; ^10^ Southern Cross University National Marine Science Centre Coffs Harbour New South Wales Australia; ^11^ Laboratoire d’Ecogéochimie des Environnements Benthiques, LECOB, Observatoire Océanologique de Banyuls sur Mer Centre National de la Recherche Scientifique (CNRS) ‐ Sorbonne Université Banyuls‐sur‐Mer France

**Keywords:** cryptic lineages, dispersal strategy, ecological speciation, life‐history divergence, mesophotic octocorals, prezygotic isolation

## Abstract

Species delimitation is central to understanding biodiversity and its conservation, yet genetic divergence among sister lineages is often insufficient to demonstrate reproductive isolation or to resolve speciation unambiguously. This limitation is especially pronounced in marine invertebrates such as corals. Their slowly evolving genomes, cryptic diversity, and complex reproductive traits can obscure species boundaries. These challenges also characterize the *Paramuricea*‐species complex occurring in the Iberian‐Atlantic, whose members, including those studied here, commonly dominate deep coral forests. Within this complex, sympatrically occurring yellow and purple morphs exhibit little mitochondrial differentiation, despite evidence of partial genetic structuring. By characterizing gametogenesis, spawning time, and early life‐history stages, we reveal pronounced prezygotic barriers between the yellow (broadcast spawning) and purple (surface‐brooding) morphs, including consistent multi‐year temporal separation and strongly contrasting fertilization environments. These differences extend into embryonic development, larval morphology, behavior, and settlement dynamics and reflect divergent dispersal strategies. Overall, our results provide direct biological evidence that the two morphs represent independent evolutionary lineages, supporting their recognition as distinct species. This model system represents a contemporary example of ecological speciation and may serve as a powerful model for future research on the genetic coupling between reproductive modes and life‐history traits.

## Graphical abstract

The two color morphs of the Iberian–Atlantic Paramuricea complex show little genetic divergence, and their species boundaries have remained unclear. By analyzing gametogenesis, spawning timing, fertilization environment, and early life‐history traits, we reveal strong prezygotic barriers and divergent developmental and dispersal strategies between the yellow broadcast spawner and the purple surface brooder. These findings provide direct biological evidence that the morphs are independent evolutionary lineages and should be recognized as distinct species.

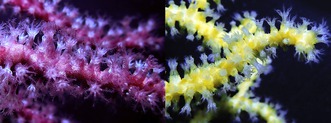

## 1 Introduction

The species is one of the key units to characterize biodiversity, and the delineation of species boundaries is fundamental to evolutionary biology and conservation (Cracraft 1989; Eric et al. 2015; Prada et al. 2008). Accurate species delimitation is not only central for understanding biodiversity patterns but also essential for identifying threatened species and formulating effective conservation strategies (Agapow et al. 2004; Bickford et al. 2007; Dillon and Fjeldså 2005). Despite significant advances in molecular methods over recent decades, which have driven major revisions in taxonomy and species identification as well as debates on the concept of species (see for example Cene et al. 2018; Cracraft 1987; De Queiroz 2007; Pinzón et al. 2013), our fundamental understanding of species as reproductively isolated entities remains largely unchanged to date.

Within the Biological Species Concept, species are then defined by the presence of barriers that limit or prevent gene flow, either before fertilization (prezygotic isolation, for example, differences in reproductive timing, habitat use, or gamete compatibility) or after fertilization (postzygotic isolation, e.g., hybrid unviability or sterility) (Coyne and Orr 1998; Mayr 1942). Speciation is, therefore, the evolutionary and dynamic process by which populations become reproductively isolated and follow independent evolutionary trajectories (Hey 2011).

Reproductive isolation is rarely measured directly, especially in marine organisms, where complex life cycles, cryptic diversity, and limited access to natural resources often complicate direct assessments. Instead, genetic divergence is frequently used as a proxy for species boundaries and species hypothesis. Genome‐wide studies generally reveal a broad correlation between the accumulation of genetic divergence and reproductive isolation, but the relationship is not straightforward (Cruickshank and Hahn 2014; Funk and Omland 2003). Deep genetic partitions may result from population structure, local adaptation, or introgression rather than true speciation (Degnan and Rosenberg 2009; Edelman et al. 2019; Naciri and Linder 2015; Sukumaran and Knowles 2017). Conversely, strong isolating barriers can arise from a limited number of loci long before genome‐wide differentiation becomes evident, especially during or shortly after speciation (Noor et al. 2001; Nosil and Feder 2012; Turner et al. 2005; Wu 2001). Where gene flow is reduced but not absent, genetic data alone can be ambiguous with respect to species status, that is, the “grey zone of speciation” (Baack and Rieseberg 2007; Gagnaire et al. 2013; Martin et al. 2013; Roux et al. 2016). While genome‐wide approaches to species delimitation are still in their early stages, integrating them with ecological and phenotypic perspectives promises to deepen our understanding of the processes linking genetic divergence to reproductive isolation (Sukumaran and Knowles 2017). Yet, such datasets remain scarce, especially for the marine environment.

The limitations of the genetic approach to species delimitation are particularly evident in sessile invertebrates such as corals, where not only phenotypic plasticity and cryptic diversity are widespread (Forsman et al. 2009; Peter 2008), but also where the exceptionally slow‐evolving mitochondrial genome of anthozoans further complicates genetic differentiation (McFadden et al. 2011; Shearer et al. 2002). This challenge is compounded by their complex reproductive biology. Corals reproduce in two primary modes: either by broadcast spawning through the release of gametes for external fertilization or by (surface) brooding, in which females retain eggs, either inside the polyp or on the external surface, but are fertilized by broadcast sperm (Brazeau and Lasker 1990; Coma et al. 1995; Harrison and Wallace 1990; Kahng et al. 2011). The reproductive strategy not only influences prezygotic reproductive compatibility but is often reflected in dispersal potential and patterns of gene flow (Cowen and Sponaugle 2009; Levitan et al. 2004). Taken together, this provides insight into the mechanisms underlying lineage divergence.

However, especially in marine broadcast spawners, closely related species that spawn within the same spatial and temporal window may hybridize, which blurs species boundaries and complicates taxonomic distinctions (Combosch et al. 2008; Quattrini et al. 2019; Vollmer and Palumbi 2002).

The octocoral genus *Paramuricea* is of considerable evolutionary interest; however, the genetic and morphological identification of its constituent species is challenging, complicating species delimitation and evolutionary inference. These gorgonians are globally distributed and predominantly inhabit mesophotic environments (Doughty et al. 2014; Grasshoff 1977; Quattrini et al. 2022; Thoma et al. 2009). In the Mediterranean Sea and along the Atlantic coast of Portugal, *Paramuricea* spp. occur in relatively shallow waters (15–200 m). At high populations densities, they form structurally complex marine animal forests (Rossi et al. 2017), whose canopies provide refuge and shelter to hundreds of species, increasing local biodiversity (Buhl‐Mortensen et al. 2017; Cau et al. 2020).

**FIGURE 1:**
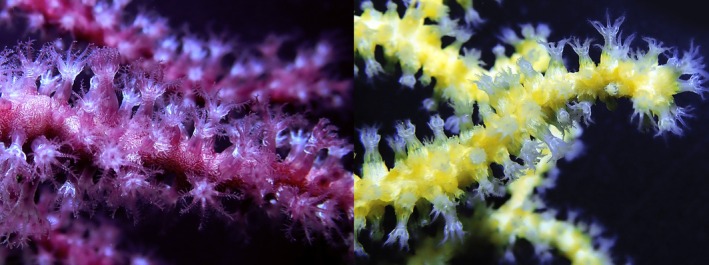
Two different color morphotypes of *Paramuricea* sp. from the SW tip of the Iberian Peninsula (Cabo de São Vicente, Sages, Portugal).

**FIGURE 2:**
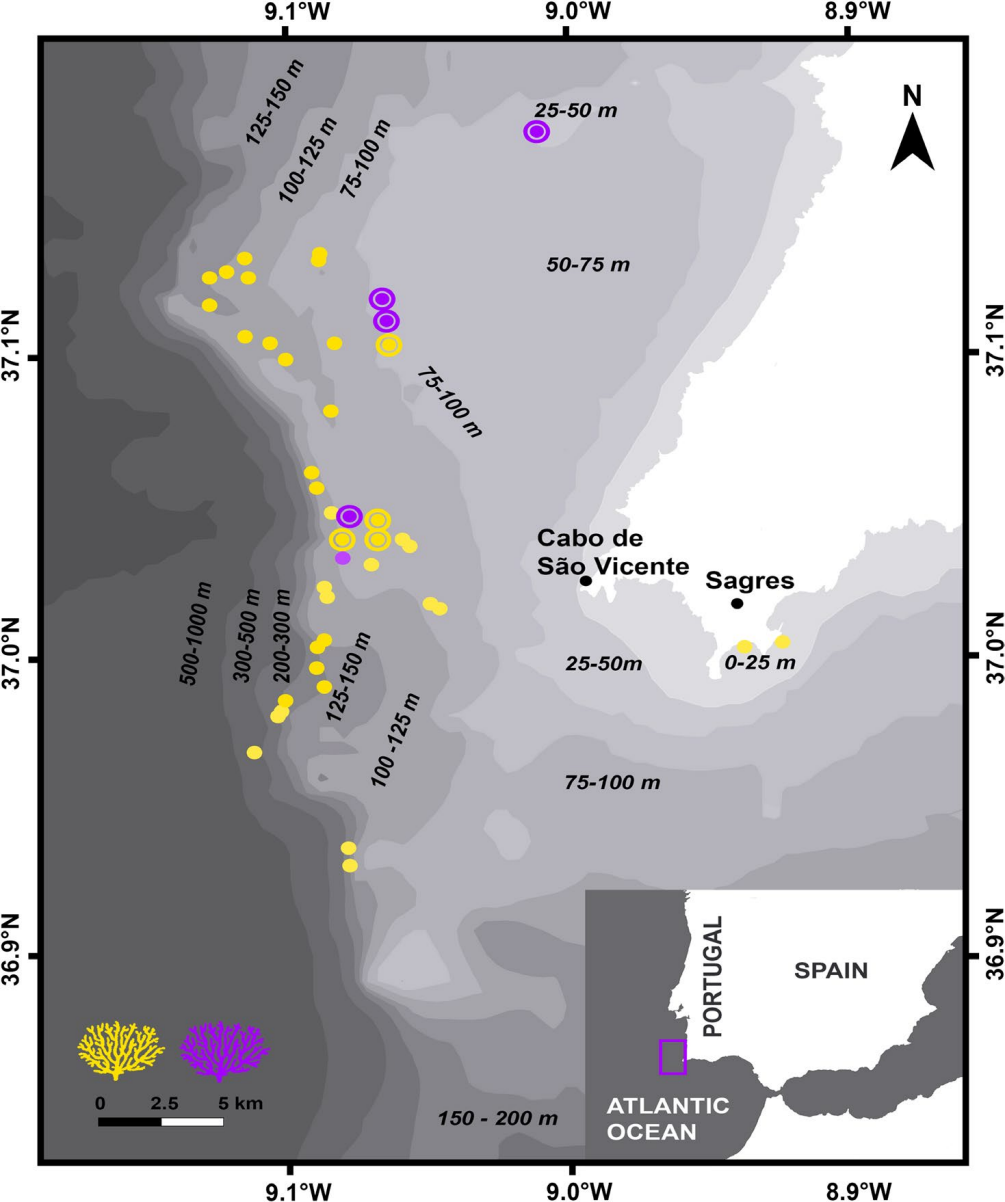
Occurrence data of yellow and purple morphotypes of *Paramuricea* sp. populations (Dias et al. 2020) and origin of the reproductive colonies in this study (double circles) at the continental shelf off Cape St. Vicent in southwestern Portugal.

Recent studies across the Atlantic‐Mediterranean transition zone, have clearly separated the Mediterranean species *Paramuricea clavata* (a well‐studied, surface‐brooding species) from an Atlantic clade exhibiting two different color morphs (Coelho et al. 2023 and Figure 1). These show some spatial segregation: the yellow morph dominates all depths along the southern Portuguese coast, where the purple morph is rare. In contrast, on the western coast, where both morphs are abundant, the purple morph predominates at shallower depths than the yellow morph (Coelho et al. 2023; Pilczynska et al. 2017; Pilczynska et al. 2019 and Figure 2). Both morphotypes occur sympatrically at a contact depth on, for example, the westernmost edge of the southern continental shelf of the Algarve (Figure 2). This shelf extends northward around the southwestern tip of the Iberian Peninsula and forms a zone known for its complex geomorphology and highly dynamic oceanographic regimes (García Lafuente and Ruiz 2007, Relvas and Barton 2002, 2005, Sánchez and Relvas 2003).

According to Coelho et al. (2022, 2023), the mitochondrial genome of the two color morphs shows little differentiation, whereas microsatellite and phylotranscriptomic analyses support some degree of divergence despite evidence of admixture. These findings raise key questions: do the yellow and purple lineages represent incipient species maintained by reproductive barriers, or are they conspecific populations structured by geography, ecology, and limited dispersal?

Because reproductive mode in octocorals is highly variable even among congeners (Kahng et al. 2011; McFadden et al. 2001), direct evidence from reproductive biology and larval traits is essential to resolve this question. By characterizing gametogenesis, spawning, and early life history in the two morphs, this study provides critical biological context for interpreting genetic divergence and evaluates whether reproductive and larval traits contribute to reproductive isolation and lineage segregation.

Specifically, this study aims to test whether differences in reproductive timing and early life‐history traits between the yellow and purple morphs of Atlantic *Paramuricea* function as mechanisms of reproductive isolation, thereby contributing to their genetic divergence and potential speciation.

## 2 Methods

### 2.1 Coral Collection and Maintenance

To investigate the reproductive traits of the two color morphs we first needed to determine their reproductive timing. To this aim we examined the presence/absence and maturation state of the gametes through both polyp dissection and histological sections (Appendix 1) from yellow morph samples caught as fishing bycatch around Cape St. Vincent (southern Portugal) since 2019 (see Dias et al. 2020 for details about the fishing activity and Figure 2). Our observations suggested that spawning occurs between late summer and autumn. Consequently, the experiments detailed below focused on this period in 2022, 2023, and 2024. In mid‐August 2022, specimens of both, the yellow (~50 colonies) and purple (~30 colonies) morph of *Paramuricea* sp. were collected as bycatch (Figure 2) and the colonies of the two morphs were maintained in separate tanks (150 to 100 L, respectively) in an indoor temperature‐controlled (15°C–16°C) semi‐closed system at the Ramalhete Marine Station (CCMAR, Faro, Portugal) for spawning observations. The room of the tank system was illuminated by dim white light, corresponding to the natural day and night hours. The water movement in the tanks was generated using wavemaker pumps. The corals were fed in the morning, and 1–2 h later, once the polyps were fully expanded, they were given an additional 40 g of a mixture of rotifers, copepods, red plankton, and mysis shrimp (Tropical Marine Centre) per tank. Oversized particles of the red plankton and mysis shrimp were pressed through a fine sieve to break them down before feeding. A subset of randomly chosen colonies was dissected and sexed. Perforated PVC cylinders equipped with 150‐μm mesh were attached to the tank outlets and were checked daily for the presence of gametes and/or larvae. In early September, several more colonies of the purple morph were collected and added to the tank. Based on the observations made in 2022, experimental monitoring of both morphs was repeated in 2023 and 2024. Colonies were collected between early July and mid‐August in several batches (in total, ~30 and 50 colonies of the purple and yellow morphs, respectively). Yellow and purple colonies were always kept separate.

### 2.2 Egg Release and Propagule Rearing

After confirming that both male and female colonies were present together in their respective tanks, the systems were monitored for the release of gametes or larvae. Branches from the colonies were regularly cut, dissected, and examined for the presence of ripe gametes. Sperm sacs were opened under a stereomicroscope to check for motile sperm, and spawning in female colonies was experimentally induced by dissecting sperm sacs in close proximity to female branches. For the tank with the purple morphotype, the first eggs, embryos, and larvae were observed to accumulate on the tank floor, and as such the bottom was vacuumed daily. The collected material was concentrated and sorted under a stereomicroscope, using the original tank water to allow for potential fertilization. Eggs and embryos still attached to the mother colony were gently removed with a pipette. During spawning of the yellow morphotype, the floating eggs and embryos were collected either by rinsing the outflow filters into containers or directly from the water column by pipetting or sieving. Eggs, embryos, and larvae were typically maintained in batches sorted by collection date in 1‐L plastic containers at 16°C in a temperature‐controlled room. Gentle water movement was ensured by aeration tubes releasing a few air bubbles per second. Approximately two‐thirds of the water was exchanged every other day. The containers were shaded with dark plastic foil to reduce excessive light penetration without keeping them completely dark.

For the offspring of the yellow morphotype, additional small 1‐L tanks were installed and supplied with system seawater in open circuit. Larvae were prevented from being flushed out by a fine mesh (150 μm) at the outflow. Water flow was kept very low to avoid larvae becoming trapped on the mesh.

### 2.3 Qualitative Developmental Observations

Different stages of embryonic and larval development were observed and photographed using a ZEISS Stemi 508 stereo microscope equipped with a ZEISS Axiocam 208 color camera system. Qualitative observations of developmental stage, shape, motility, and settlement were conducted on > 10 batches of 50–600 larvae each throughout the three reproductive events in 2022, 2023, and 2024. Samples at each stage of development were prepared for scanning electron microscopy (SEM). The samples were fixed overnight at 4°C in 4% glutaraldehyde buffered in 0.1–0.5 M Soerenson's phosphate buffer. The concentration was adjusted according to larval buoyancy of each developmental stage (molarity was decreased if larvae floated or increased if they sank). The following day, the propagules were washed in pure buffer and transferred into 30% ethanol. All samples were transferred to the SNSB Zoological State Collection in Munich (Germany), where they were dehydrated through a graded series of acetone solutions (30%, 50%, 75%, 95%, and 100%, soaked for 10 min at each step, and two final rinses in 100% acetone). Samples were then dried in a critical point dryer (BioRAD Model E4850 refrigerated recirculatory), transferred onto SEM stubs covered with self‐adhesive carbon stickers and gold coated in an argon atmosphere using a Polaron SC510. Three embryos of each stage of development were then analyzed with a LEO 1430 VP SEM at 15 kV.

### 2.4 Quantitative Embryogenesis

In 2023, immediately after gamete release was observed, female sexual products were collected with tank water and left for 30 min to 1 h to ensure fertilization. They were then counted and transferred into 6‐well plates, still in the original tank water. The number of replicates and female sexual products per replicate varied depending on availability of the yellow and purple morph: for the purple morph, five replicates of 50 propagules each were monitored, while for the yellow morph, 2 replicates of 100 propagules each were used. The plates were checked every 10 min for the onset of cleavage.

Once the first cleavage occurred, each well containing embryos, corresponding to one replicate, was photographed in several overlapping images using a stereomicroscope at 8× magnification over millimeter paper. The photographing intervals were adapted to cleavage rate (every 10 or 20 min for the yellow morph and every 30 or 40 min for the purple morph). The images were later analyzed using the Cell‐Count plugin in ImageJ‐Fiji for counting. Embryos were categorized and counted according to their developmental stages: 1‐cell, 4‐cell, 8‐cell, 16‐cell, 32‐cell, 64‐cell, blastula, gastrula, or larvae.

### 2.5 Larval Shape

The larval length‐to‐width ratio was measured from day 3 of larval age until day 15 for both morphs. The measurements were obtained from images of moving larvae from four cohorts of the yellow morph and five cohorts of the purple morph. The pictures were taken randomly to include a representative fraction of larvae from each cohort. The images were edited in Photoshop 22.2.0 to enhance the contrast and clarify the edges of the larvae. The scale was set, and the length and width of the larvae were measured using the measurement tool in Photoshop and recorded. Only larvae with visible edges and those swimming in the *x* or *y* direction (not in the *z* axis) were included in the measurements. The length‐to‐width ratio was calculated and the effects of species, age (days post‐spawning), and their interaction on larval length‐to‐width ratio using a linear model (lm in R) were analyzed. Species and age were treated as fixed effects, and model assumptions were verified by inspection of residuals. Significance of main and interaction effects was assessed using Type I ANOVA.

### 2.6 Onset of Swimming and Swimming Behavior

Videos of embryo/larval behavior were recorded daily from day 1 to day 20 for both morphs. For the yellow morph, four cohorts (Sept 28th, Sept 29th, Sept 30th, Oct 1st) were tracked in 2024, capturing approximately 2 videos of 10 larvae per cohort, recorded for 10 min each, when available. Larvae were randomly selected from the cohorts and carefully placed into a 10 × 10 × 1 cm box with a black background and millimeter scale. When fewer than 20 larvae were available per cohort (e.g., due to mortality with age), all remaining larvae were recorded in later stages.

For the purple morph, videos were recorded for three different cohorts (Aug 2nd, Aug 3rd, and Sept 3rd in 2024). Larvae were randomly selected, and then carefully placed into a 10 × 10 × 1 cm box with millimeter‐paper background and their behavior was captured for 3 min. As larvae from the batches died over time, fewer individuals remained for recording of later stages.

Swimming behavior was analyzed from the first 3 min of each video. Because distinguishing active swimming from passive floating or sinking was not always possible, these were grouped into ‘upward’ (surface or ascending) and ‘downward’ (bottom or descending) movements. From raw counts, the percentages of upward‐ and downward‐moving larvae were calculated, along with mean, standard deviation, and standard error for each morph and day. A Wilcoxon rank‐sum test (Mann–Whitney U test) was performed in R Studio 2024.12.1 to test for significant differences in overall swimming‐up behavior by species.

### 2.7 Swimming Speed

Swimming speed was quantified using the same video recordings as for the swimming behavior analysis (see above). Based on qualitative observations indicating differences between younger and older larvae, recordings from 4‐ and 12‐day‐old larvae were selected. Each video represented one replicate containing 5–9 individuals. Swimming speed was calculated by measuring the distance traveled over time over the background millimeter scale for clearly visible, actively swimming larvae and mean velocities from a total of 25 measurements from 4‐day old yellow larvae, 18 measurements for 12‐day‐old yellow larvae and 16 measurements of 12‐day‐old purple larvae, were computed. Active swimming was considered when yellow larvae exhibited typical small zigzag movements (Video 2), whereas purple larvae rotated counterclockwise around their longitudinal axis while swimming upward (Video 6). Purple 4‐day‐old larvae did not show active upwards swimming.

### 2.8 Onset of Settlement

The onset and progression of settlement over time were determined from several different settlement experiments conducted across the 3 years. Substrates that induced settlement in earlier trials were added at various times to assess if settlement depends on the timing of substrate encounter. For the purple morph of *Paramuricea* sp., a settlement experiment was conducted in 2024, in which 100 larvae (collected on September 3, 2024) were offered different substrates (CCA, black rock and brown rock, all from fisheries bycatch) at the age of 5 days, 10 days, and 15 days. The other two replicates (collected on August 3, 2024 and August 5, 2024) were excluded due to handling errors.

For the yellow morph, larvae were collected on October 25, 2022 (50 larvae, 5 replicates), and September 30, 2023 (50 larvae, 4 replicates). The 2022 cohort was exposed on day 5 to gorgonian skeletons, which were the only settlement‐inducing substrates available at the time, whereas the 2023 cohort was exposed on day 15 to gorgonian skeletons, but also to CCA, and rock (all from fisheries bycatch).

## 3 Results

### 3.1 Sex Ratio

In 2023 and 2024, a total of 98 randomly sampled colonies from the yellow morph were sexed with 33 females (34%) and 65 male colonies (66%), resulting in a male‐to‐female ratio of 1.97 to 1. Of the purple morph, 87 colonies were sexed into 32 females (38%) and 55 males (63%), corresponding to a male‐to‐female ratio of 1.72 to 1.

### 3.2 Timing of Reproduction

#### 3.2.1 Purple Morphotype

**FIGURE 3:**
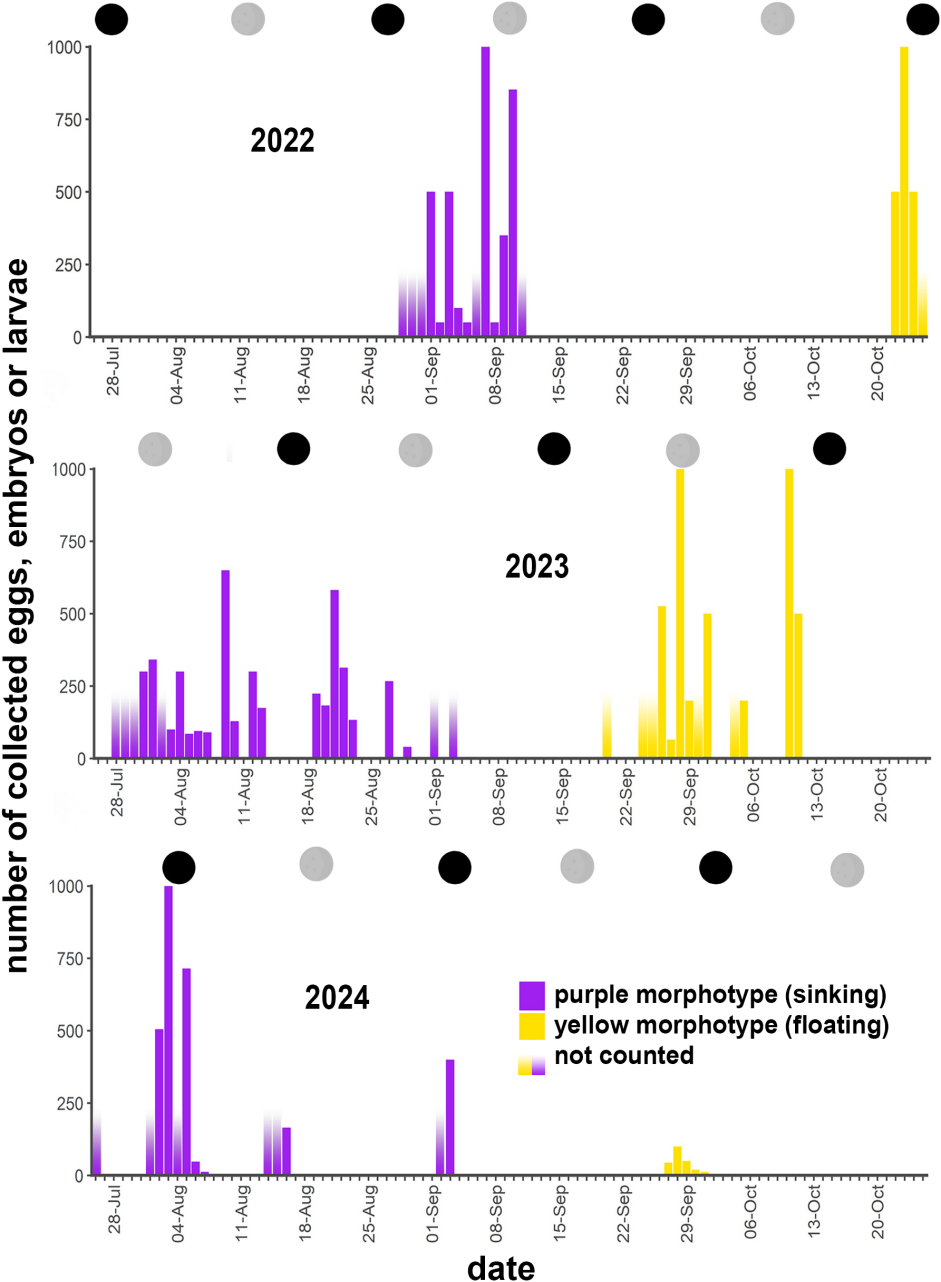
Reproductive timing of purple and yellow morphs of *Paramuricea* sp. in 2022, 2023, and 2024 related to the moon cycle. The number of female sexual products that were collected from the tank floor (i.e., negatively buoyant, purple color) or from the overflow/water column (i.e., positively buoyant, yellow color) per spawning day is indicated. Circles in the top rows represent the moon phases, full moon (in gray) and new moon (in black).

On September 1st 2022, we made our first observations of propagules of the purple morph on the tank floor. As a part of them was already moving, the first release most likely occurred on the evening of August 29, 2022 or during early morning of August 30, 2022. The colonies continued releasing over the following 10 days, including those brought in freshly from the field in early September. In 2023, the spawning of the purple colonies began about 1 month earlier, starting on July 28 and occurred regularly every day or every other day over a whole month until August 27, 2023 (Figure 3). The polyps of colonies collected from the field on August 25, 2023 were either already empty or had few remaining gametes, which they released over two consecutive days while the conspecifics were already in the tanks, indicating a natural and synchronized spawning with the wild populations. A correlation with the moon cycle was not obvious (Figure 3).

#### 3.2.2 Yellow Morphotype

In colonies of the yellow morph, we observed the first release of female sexual products in the tanks during the night from October 21 to 22, 2022. This occurred more than a month later than the last observed spawning of the purple colonies. We could then repeatedly observe spawning over nearly a month at irregular intervals until November 25, 2022. In 2023, spawning occurred from September 21, 2023 until October 11, 2023, again several weeks after the purple colonies had ceased releasing (Figure 3). Peak release days occurred during or shortly before the full or new moon. When spawning was observed shortly before the new moon the reproductive window was much narrower, however, clear evidence for a correlation is still lacking.

### 3.3 Mode of Reproduction

#### 3.3.1 Purple Morphotype

**FIGURE 4:**
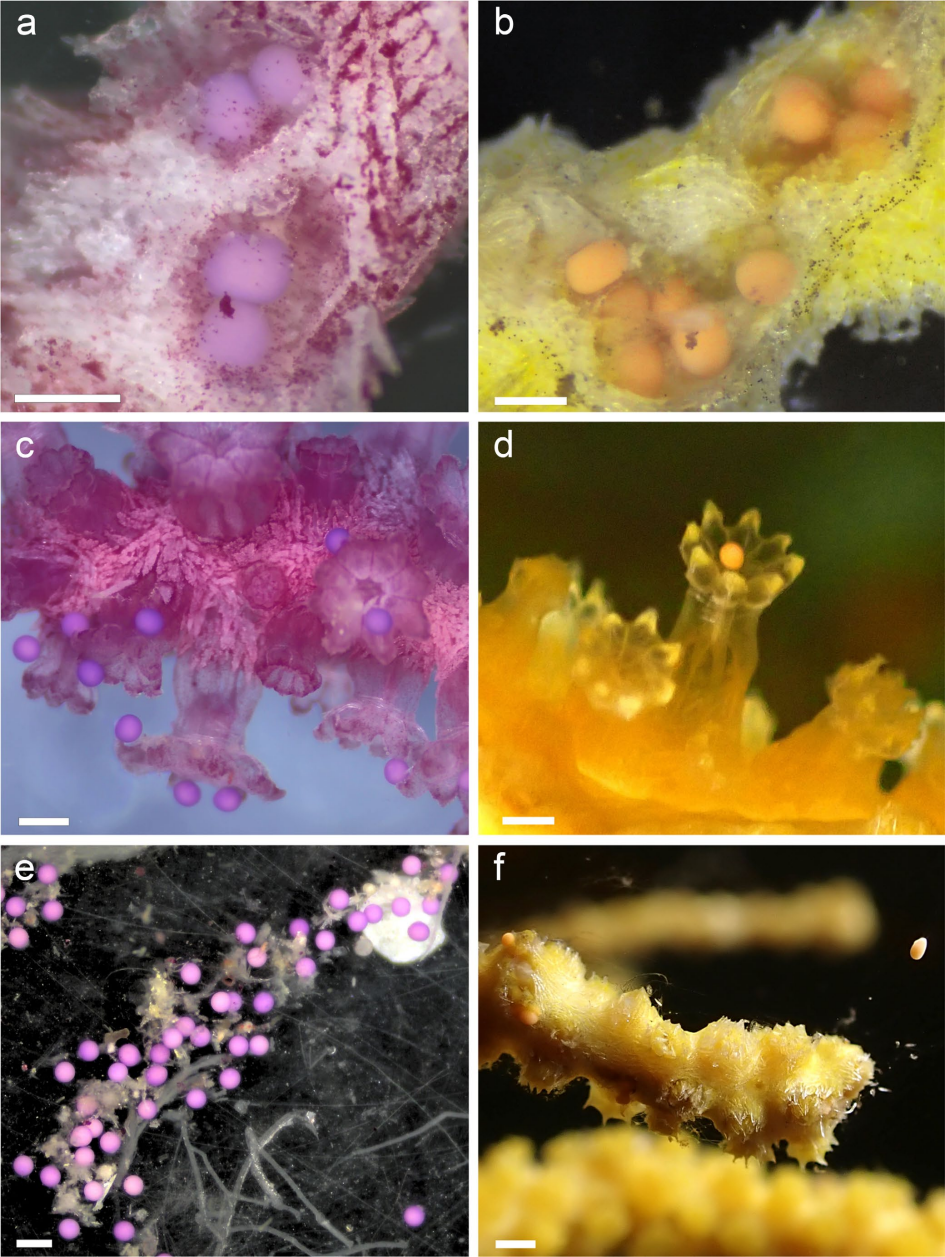
Reproductive phenology of *Paramuricea* sp. purple (a, c, and e) and yellow (b, d, and f) morphotypes. Ripe oocytes of the purple morph are visible after dissection of the polyps (a), egg release, staying on the mother colony (c), before clustering in mucus strings on the tank floor (e). Ripe oocytes of the yellow morph are visible after opening the tissue (b), egg release (e), and a floating egg in the water column (f). Scale bars = 500 μm.

**FIGURE 5:**
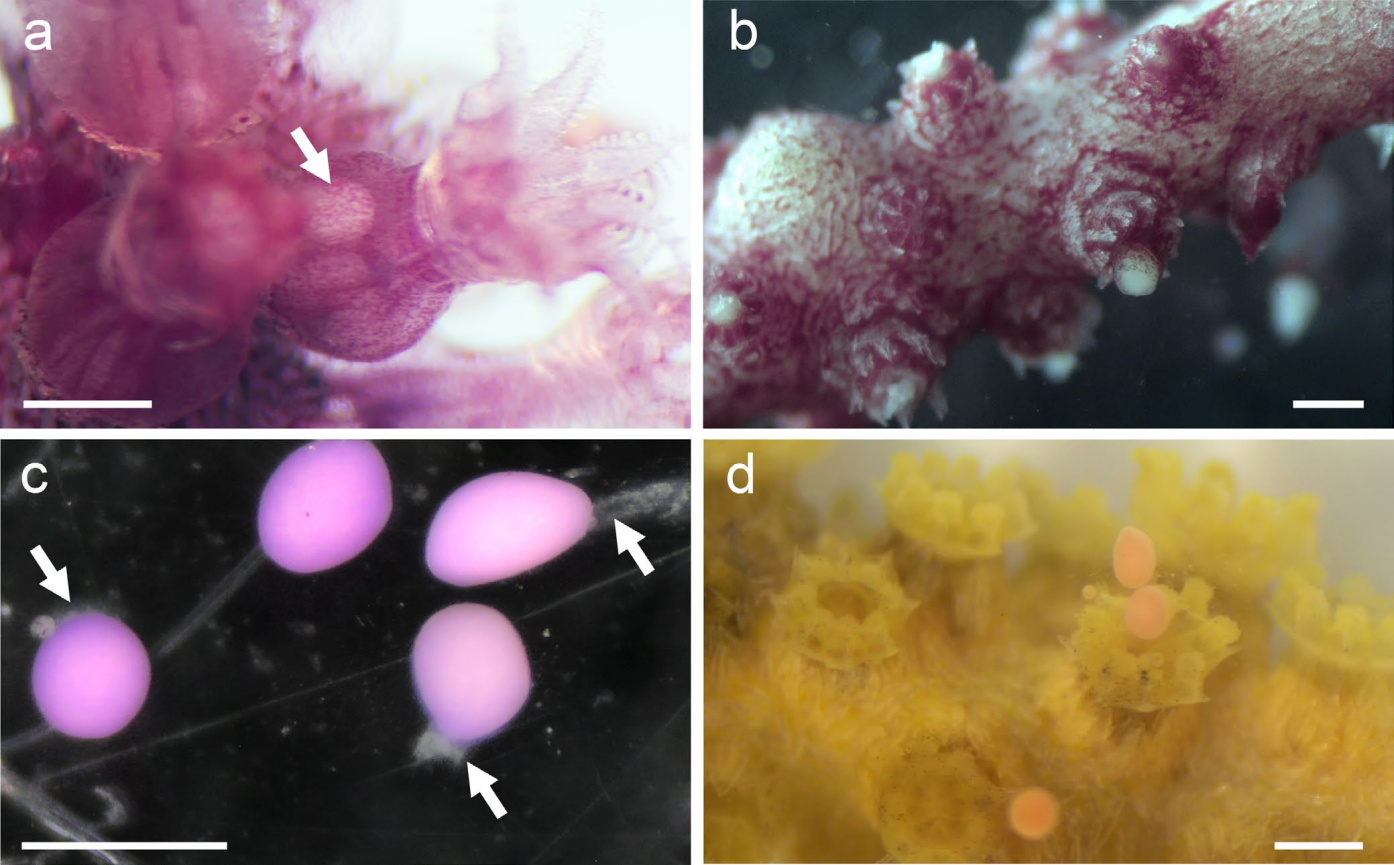
Reproductive phenology of *Paramuricea* sp. purple (a–c) and yellow (d) morphotypes. Sperm bundles move upward within the polyps of the purple morphotype (a) and are released before rupturing in response to stronger water movement (b). During fertilization, dense sperm clouds form at one pole of the egg (c). Female spawning was induced in a branch of the yellow morphotype by dissection of ripe male gonads (d). Scale bars = 500 μm.

Colonies of the purple morphotype reproduced by surface brooding. In general, egg release was slow and directly observable, occurring both during the day and at night (see Video 1). The synchronic release occurred at the branch level, with polyps of the same branch releasing eggs at the same time while different branches releasing at different moments. Additionally, not all eggs in a polyp were released at the same time so that the same branch released multiple times over several days, alternating with the other branches. All collected female sexual products were negatively buoyant and surrounded by adhesive mucus when released, forming string‐like assemblages of clustered propagules (Figure 4e) that initially remained on the surface of the colony (Figure 4b) and then sank. If current was present, the mucus strings drifted with the current until reaching the floor or becoming entangled. Natural sperm release was not observed in the tanks. However, cut branches from male colonies released sperm bundles when kept together with recently spawned females in small containers under a stereomicroscope (Figure 5a–c). The sperm bundles slowly moved upward within the polyps (Figure 5a) and opened and dissolved outside the polyp in response to strong water movement (Figure 5b). Fertilization was observed when sperm accumulated and formed dense clouds on one side of the eggs (Figure 5c). Female spawning could not be induced by opening ripe male gonads; however, the female colonies may not have been sufficiently ripe.

**VIDEO 1:** Oocyte release in *Paramuricea* sp. purple morphotype. Direct observation of oocyte release in the purple morphotype of *Paramuricea* sp. during daytime. Reproductive output occurs in a surface‐brooding mode, with oocytes slowly released from the colony surface and remaining close to the mother colony. Video content can be viewed at https://onlinelibrary.wiley.com/doi/10.1002/ece3.73264.

#### 3.3.2 Yellow Morphotype

Colonies of the yellow morphotype reproduced by broadcast spawning. Similar to the colonies of the purple morph, the egg release in the yellow morph occurred slowly during both day and night. The female sexual products were floating in the tank and eventually accumulated in the overflow filters, from where they were collected. None of the collected female sexual products was adhesive, and no signs of mucus were visible on either the products or the colonies (Figure 4f). Sperm release in the tanks was also not observed in yellow colonies, however, egg spawning could be induced by opening ripe sperm bundles next to female branches in a Petri dish (Figure 5d).

### 3.4 Embryogenesis

#### 3.4.1 Purple Morphotype

**FIGURE 6:**
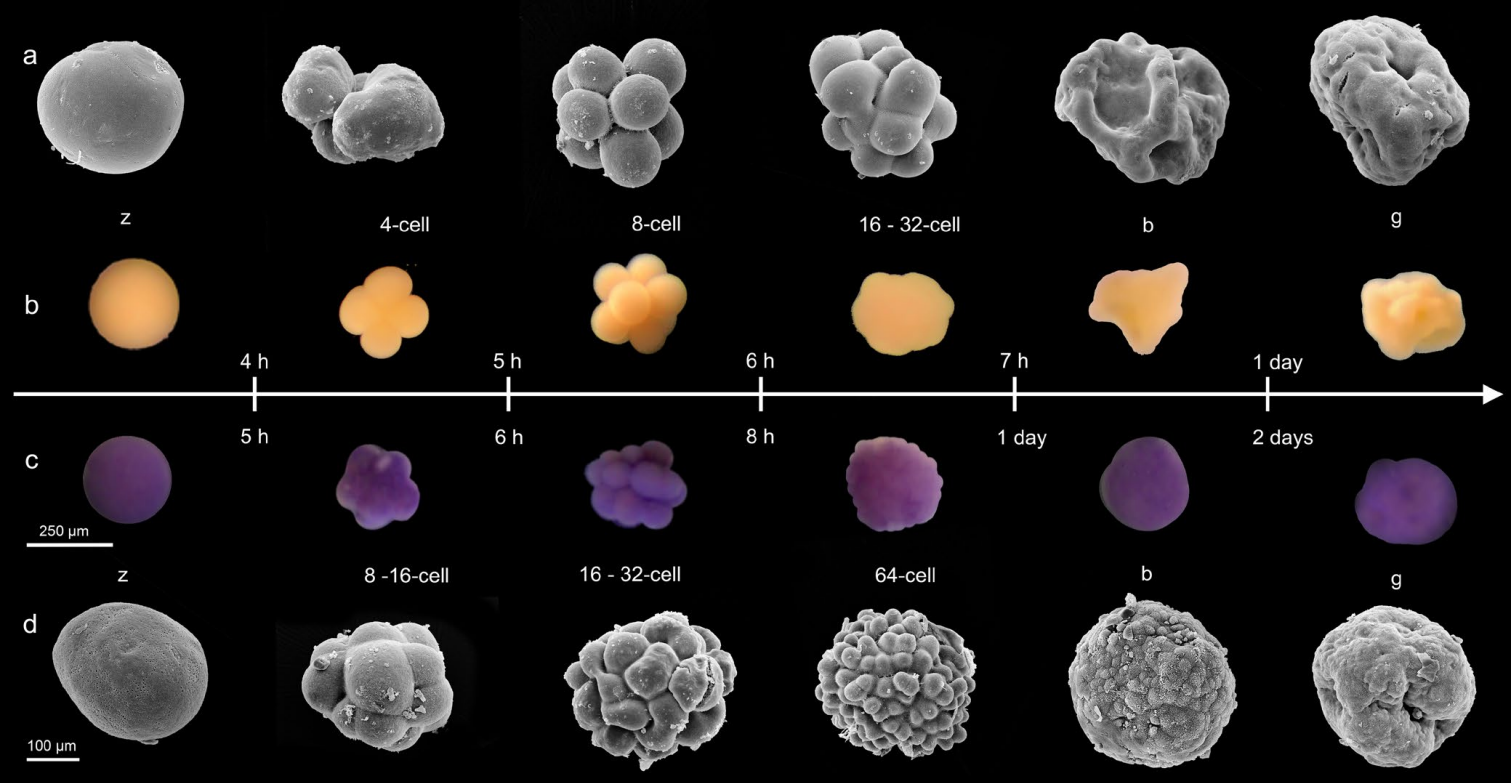
Scanning electron microscopy (SEM) micrographs and light microscopic (LM) development timeline of embryos of the yellow and purple *Paramuricea* sp. morphotypes. *Paramuricea* sp. yellow morph: SEM micrographs of the zygote, 4‐cell‐, 8‐cell‐, 16‐cell‐stage embryo, blastula, and gastrula (a) and LM captures of the zygote, 4‐cell‐, 8‐cell‐, 32‐cell‐stage embryo, blastula, and gastrula (b). *Paramuricea* sp. purple morph: LM captures of the zygote, 8‐cell‐, 16‐cell‐, 64‐cell‐stage embryo, blastula, and gastrula (c). SEM micrographs of the zygote, 8‐ to 16‐cell‐, 32‐cell‐, 64‐cell‐stage embryo, blastula, and gastrula (d). b, blastula; g, gastrula; z, zygote. The scale applies for all LM and SEM micrographs.

**FIGURE 7:**
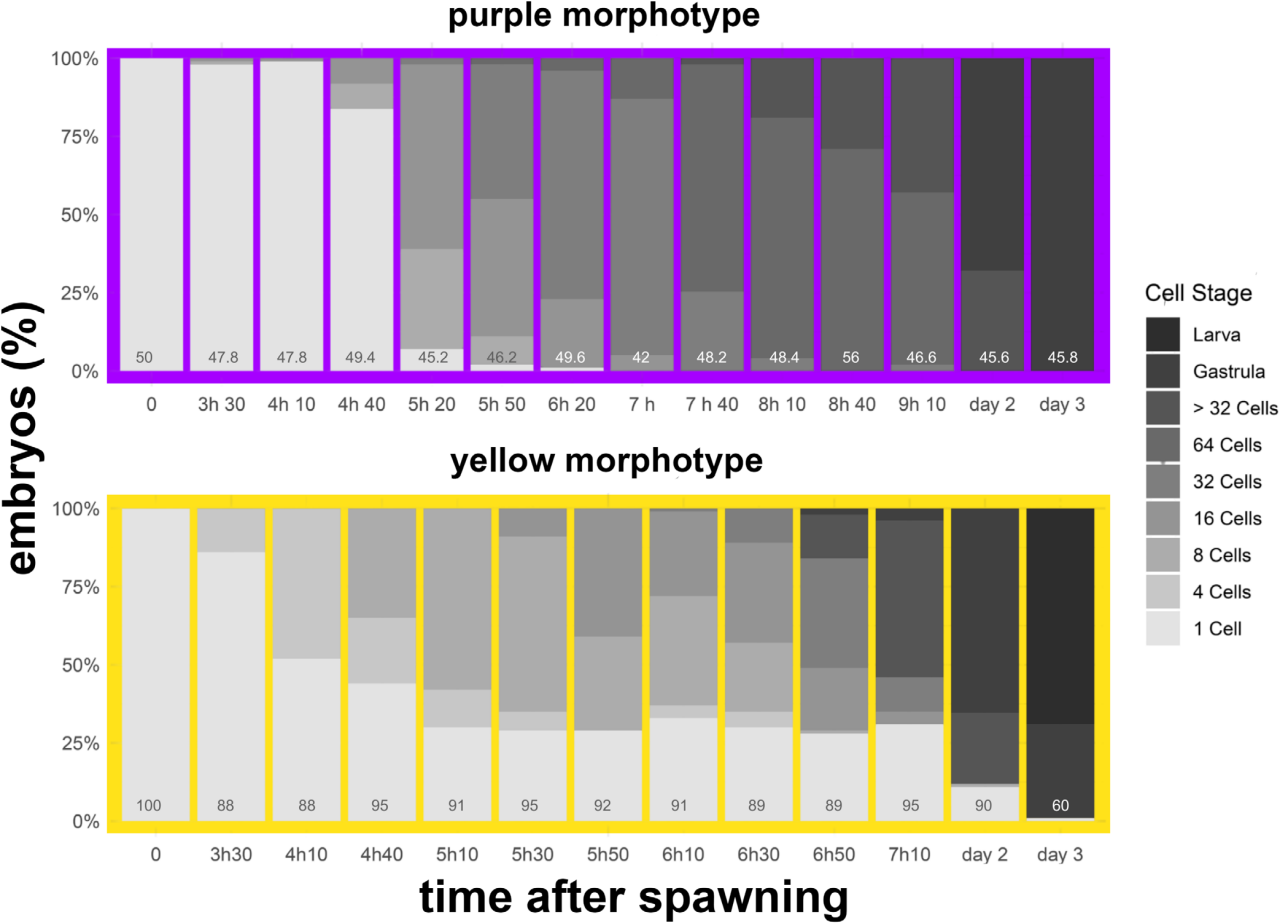
Differences in embryonic development of *Paramuricea* sp. purple (upper panel) and yellow (lower panel) morphotypes. Proportion of embryos in each development stage over 3 days post spawning. Purple morphotype: Average percentage out of 5 replicates of 50 eggs each. Yellow morphotype: Average percentage out of 2 replicates, 100 eggs each. Numbers on the bottom of the bars indicate the average total number of embryos at the timepoint.

The first cleavage of fertilized eggs from the purple morph was observed 3 h and 40 min after fertilization. Cleavage was clearly visible for 8‐cell and 16‐cell embryos, whereas 2‐cell and 4‐cell stages were barely discernible. The cell bond appeared comparatively tight (Figure 6d, 8 to 16‐cell). Detachment of individual cells was barely observed. Between 5 and 5.5 h, over 50% of the embryos cleaved into 16‐cell embryos, and by 6 to 6.5 h, the embryos reached the 32‐cell stage (Figure 6d 16 to 32‐cell, Figure 7). All monitored eggs developed, indicating a 100% fertilization rate. Between 7 and 7.5 h, most embryos reached the 64‐cell stage (Figure 6d, 64‐cell, Figure 7). The cell bond appeared looser than those of 8‐ to 16‐cell embryos, and detachment of single cells was observed in most embryos. From 7.5 to 24 h, embryos developed into blastulas (Figure 6b, Figure 7) and subsequently underwent gastrulation. After 48 h, the embryos remained in the gastrula stage (Figure 7).

#### 3.4.2 Yellow Morphotype

**FIGURE 8:**
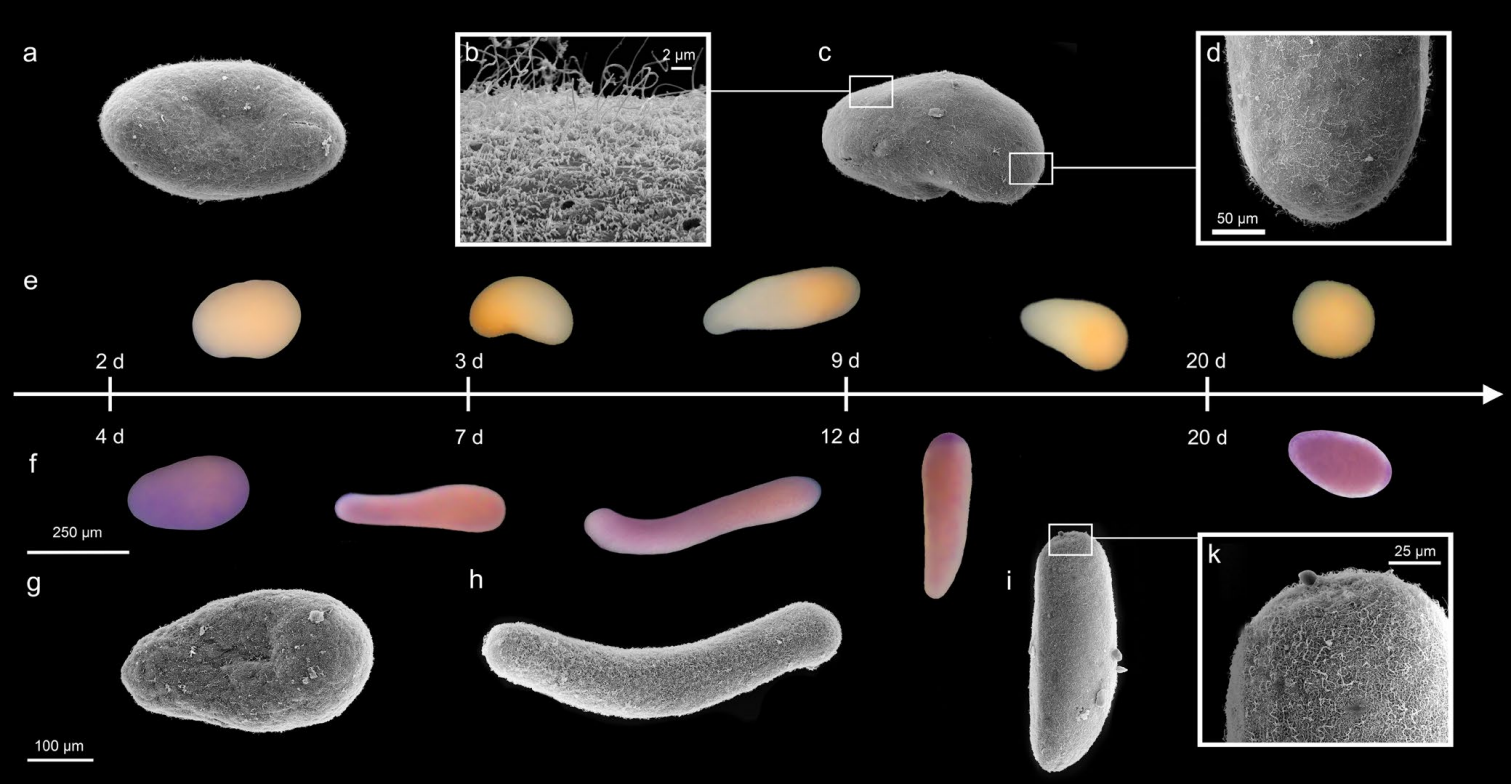
Scanning electron microscopy (SEM) micrographs and light microscopic (LM) development timeline of larvae of the yellow and purple *Paramuricea* sp. morphs. a‐e *P*. sp. yellow morph: SEM micrographs of a 2‐day old larva (a), the flagella and surface pores (b) of 9‐day old larvae (c) and its aboral pole (d) and LM pictures of 2 to over 20 days old larvae (e). (f–k) *P*. sp. purple morph: LM pictures of 4 days to over 20 days old larvae (f) and SEM micrographs of the 4‐day old larvae (g), 8‐day old larva (h) and 15‐day old larva (i) and its aboral pole (k).

In zygotes of colonies of the yellow *Paramuricea* morph, the first cleavage occurred around 3.5 h after collection. Approximately 4.5–5 h later, nearly 50% of the zygotes followed had cleaved into 4‐cell embryos, and an hour later, more than 50% progressed to the 8‐cell stage (see Figure 7). The blastomeres well‐demarcated (Figure 6a, 4‐cell and 8‐cell) and embryos frequently disaggregated (Figure 6a, 4‐cell). The individual cells, however, were observed to continue development leading to an increased count of developing embryos (see supplement). By 5.5–6 h, about one‐third of the embryos reached the 16‐cell stage. Few embryos reached the 32‐cell stage (7, Figure 6b, 16 to 32‐cell), and a distinct 64‐cell stage was not observed. Instead, the embryos became increasingly irregular in shape, with distinct cells no longer visible beyond the 16–32 cell stage (Figure 6b). Over 25% of the eggs did not develop within the first 7.5 h (see Figure 7). Between 7.5 and 24 h, most embryos entered the gastrula phase (Figure 6g). By 48 h, the embryos had already developed into early larvae and began swimming (Figure 8a,e).

### 3.5 Larval Morphology

**FIGURE 9:**
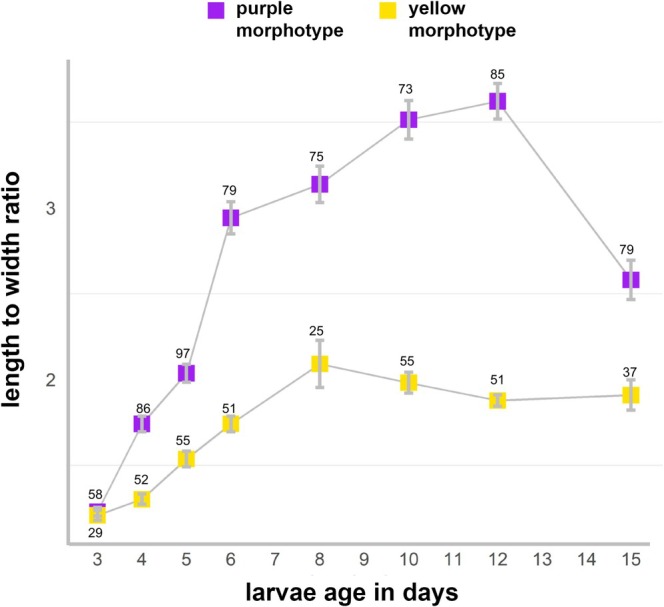
Differences in larval size of the two morphotypes of *Paramuricea* sp. Mean length to width ratio (± SE) of the purple and the yellow morph in relation to larval age. Numbers above the bars indicate the number of individuals measured for each time point.

The linear model showed significant effects of species, age, and their interaction on the larval length‐to‐width ratio. The yellow morph exhibited lower ratios than the purple morph (estimate = −0.48 ± 0.13 SE, *p* < 0.001 lower than purple larvae), and the ratio increased with age (estimate = 0.116 ± 0.008 SE, *p* < 0.001). A significant species × age interaction (estimate = −0.057 ± 0.015 SE, *p* < 0.001) indicated that changes in larval shape with age were not the same in both morphotypes, with the yellow morph elongating more slowly over time. The ANOVA confirmed strong species (F_1,1182_ = 325.10, *p* < 0.001), age (F_1,1182_ = 230.91, *p* < 0.001), and interaction effects (F_1,1182_ = 15.52, *p* < 0.001). So, larvae of the purple morph are generally more elongated than the more roundish or oval shaped larvae of the yellow morph (compare also Figure 8a,c,c,e,f,h,i and Figure 9). Even though the shape changes with age, the difference between the two morphs remains consistent. Larvae of the purple morph not only had a greater change in length over time but also exhibited a distinctively colored and flattened aboral pole (Figure 8f,k), while the larvae of the yellow morph only showed distinction between an opaque aboral and more translucent oral region (Figure 8e).

### 3.6 Onset of Swimming and Swimming Behavior

**FIGURE 10:**
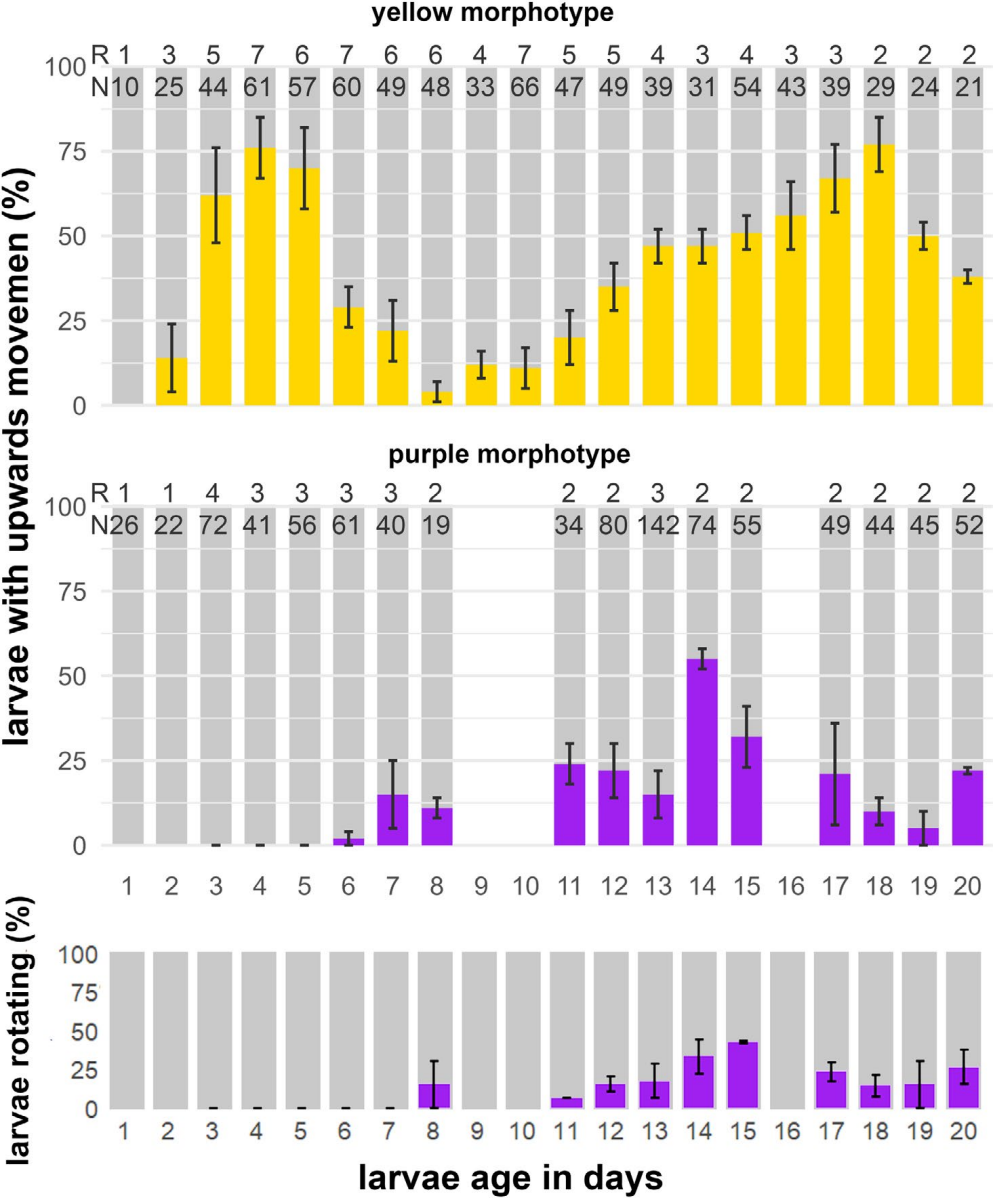
Differences in behavior of the two morphotypes of *Paramuricea* sp. (yellow and purple). Average percentage (± SE) of larval upward swimming behavior after 3 min of recording, and larvae of the purple showing rotating behavior. Numbers in the row labeled with R are the number of replicates (cohorts). The row labeled with N represents the total amount of larvae used in the experiment per age group.

The larvae of the yellow and purple *Paramuricea* morphs differed in their swimming phenology and timing of up‐ and downward swimming/floating (*W* = 821.5, *p* = 1.358e‐06; Figure 10). In the yellow morph, as we did not differentiate between upwards swimming or floating, upwards movement was observed already on day 1 after spawning (age 1 day), when they were still embryos but did not show typical swimming behavior (see Video 2). More than 50% of the larvae exhibited upward movement displaying active swimming movement from day 3 onward until day 6, when the mean upward movement behavior gradually decreased until day 8. After day 8, upward movement increased again slowly until day 18 (Figure 10). From the onset of movement, qualitative observations of swimming showed that larvae were agile, quickly changing shape and swimming direction (see Video 2 for 4 day and Video 3 for 12 day old larvae swimming). Larvae of the purple morph started moving from day 4 onwards, however they were only observed to slowly crawl over the floor (Videos 4 and 5) until larvae began upward movement on day 6 post‐spawning. Upward movement activity then increased slowly but not steadily, peaking around day 14 and declining again thereafter (Figure 10). Rotational behavior began once larvae started upward swimming and was primarily associated with upward movement, though it also occurred at the bottom (Figure 10 and Video 6, note also the clearly visible dent of the aboral pole).

**VIDEO 2:** Swimming behavior of 4‐day‐old larvae of *Paramuricea* sp. yellow morphotype. Active swimming behavior of 4‐day‐old larvae of the yellow morphotype. Larvae constantly change body shape and swimming direction while swimming rapidly and exhibit characteristic short zigzag movements in the water column. Video content can be viewed at https://onlinelibrary.wiley.com/doi/10.1002/ece3.73264.

**VIDEO 3:** Swimming behavior of 12‐day‐old larvae of *Paramuricea* sp. yellow morphotype. Twelve‐day‐old larvae of the yellow morphotype show more elongated body shapes and no longer change shape as frequently but remain agile swimmers. Video content can be viewed at https://onlinelibrary.wiley.com/doi/10.1002/ece3.73264.

**VIDEO 4:** Limited swimming activity of 4‐day‐old larvae of *Paramuricea* sp. purple morphotype. Four‐day‐old larvae of the purple morphotype show no active upward swimming. Larvae remain near the substrate and move slowly across the surface. Video content can be viewed at https://onlinelibrary.wiley.com/doi/10.1002/ece3.73264.

**VIDEO 5:** Crawling behavior of larvae of *Paramuricea* sp. purple morphotype. Larval movement of the purple morphotype observed from day 4 onward. As larvae become more elongated with age, they primarily crawl slowly along the substrate surface rather than actively swimming in the water column. Video content can be viewed at https://onlinelibrary.wiley.com/doi/10.1002/ece3.73264.

**VIDEO 6:** Rotational swimming behavior of *Paramuricea* sp. purple morphotype larvae. Upward swimming behavior of larvae of the purple morphotype characterized by counterclockwise rotation around the longitudinal body axis. Rotational movement begins once larvae initiate upward swimming and is primarily associated with vertical movement, although it can also occur near the substrate. The indentation at the aboral pole is clearly visible. Video content can be viewed at https://onlinelibrary.wiley.com/doi/10.1002/ece3.73264.

Swimming speed could not be measured on day 4 for the purple morph as no larvae were actively swimming. Yellow morph larvae swam upward at 0.40 ± 0.13 mm s^−1^ on day 4, decreasing to 0.22 ± 0.07 mm s^−1^ by day 12. In contrast, swimming purple larvae were relatively fast at 0.50 ± 0.14 mm s^−1^.

### 3.7 Onset of Settlement

**FIGURE 11:**
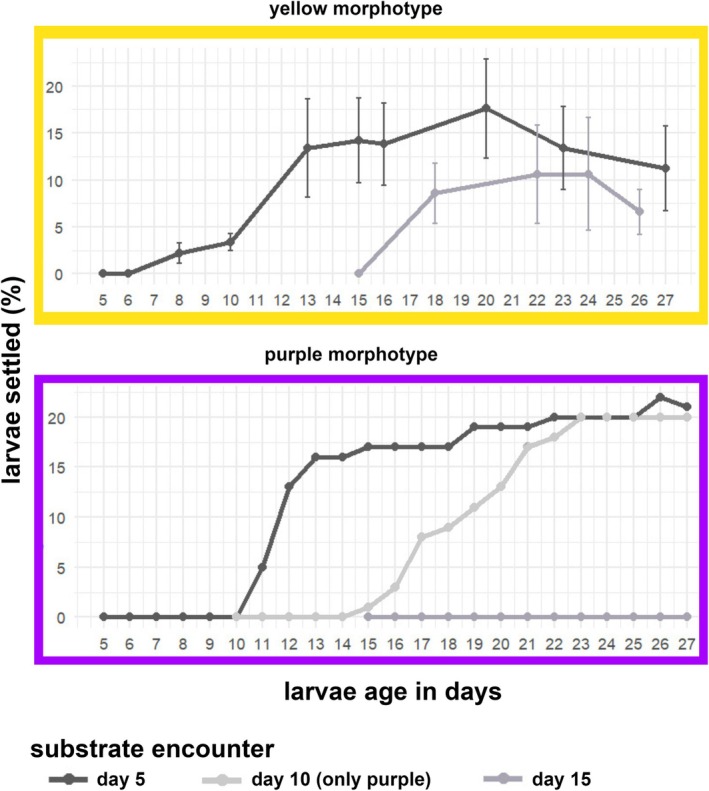
Differences in settlement dynamics of the yellow (top) and purple (bottom) morphotypes of *Paramuricea* sp. Percentage of settlers after exposing larvae to substrate at 5 (10) and 15 days of age. Yellow morphotype shows the average percentage of settlers (±SE) across 50 larvae (*N* = 50) per replicate (*n* = 5). Purple morphotype shows the percentage of settlers out of 100 larvae (*N* = 100) used for the experiment (*n* = 1).

Settlement success depended on the timing of substrate availability. When substrate was provided to the larvae of the purple morph on day 5 and 10, settlement began after 4 and 3 days, respectively (Figure 11). No settlement happened when substrate was provided on day 15. The earliest settlement was observed on day 11, and the latest on day 26, with most settlement occurring between age days 11 and 23. These results should be taken with caution as no replicates of the experiment could be evaluated. We did, however, see qualitatively similar behavior in other cohorts in all three reproductive seasons, that were not part of the experiment presented here.

The earliest onset of larval settlement of the yellow morph was observed at the age of 8 days when substrate was provided on day 5, and is consistent with observations on other cohorts. When larvae were provided with substrate on day 15, they remained competent to settle until age 22 days, similar to larvae of the purple morphs.

## 4 Discussion

### 4.1 Differences in Life‐History Traits Provide Effective Barriers to Gene Flow and Support the Speciation Hypothesis

This study underscores the central importance of ecological and reproductive–phenological data to understand speciation processes. Integration with genetic and genomic data would provide crucial insights for resolving taxonomic uncertainties and for understanding the evolutionary mechanisms underlying species divergence (Quattrini et al. 2013).

Although based on our reproductive data the lack of differentiation of mitochondrial genomes (Coelho et al. 2022, 2023) between the two color morphotypes of the *Paramuricea* complex in the Atlantic may appear surprising, it is consistent with the generally low mitochondrial diversity typical of anthozoans (Doughty et al. 2014; McFadden et al. 2011; Quattrini et al. 2013). Phylotranscriptomic analyses revealed some degree of genetic differentiation yet could not conclusively determine whether these morphs represent distinct species, particularly in sympatric populations (Coelho et al. 2023). Our observations on the reproductive ecology, strongly suggest the existence of a prezygotic isolation resulting from a mismatch of reproduction timing and mode, consistent with the biological species concept (Mayr 1942, Figure 12). Moreover, they reveal distinct contrasting reproductive between the two morphotypes, with very likely differentiation in dispersal (Figure 13).

**FIGURE 12:**
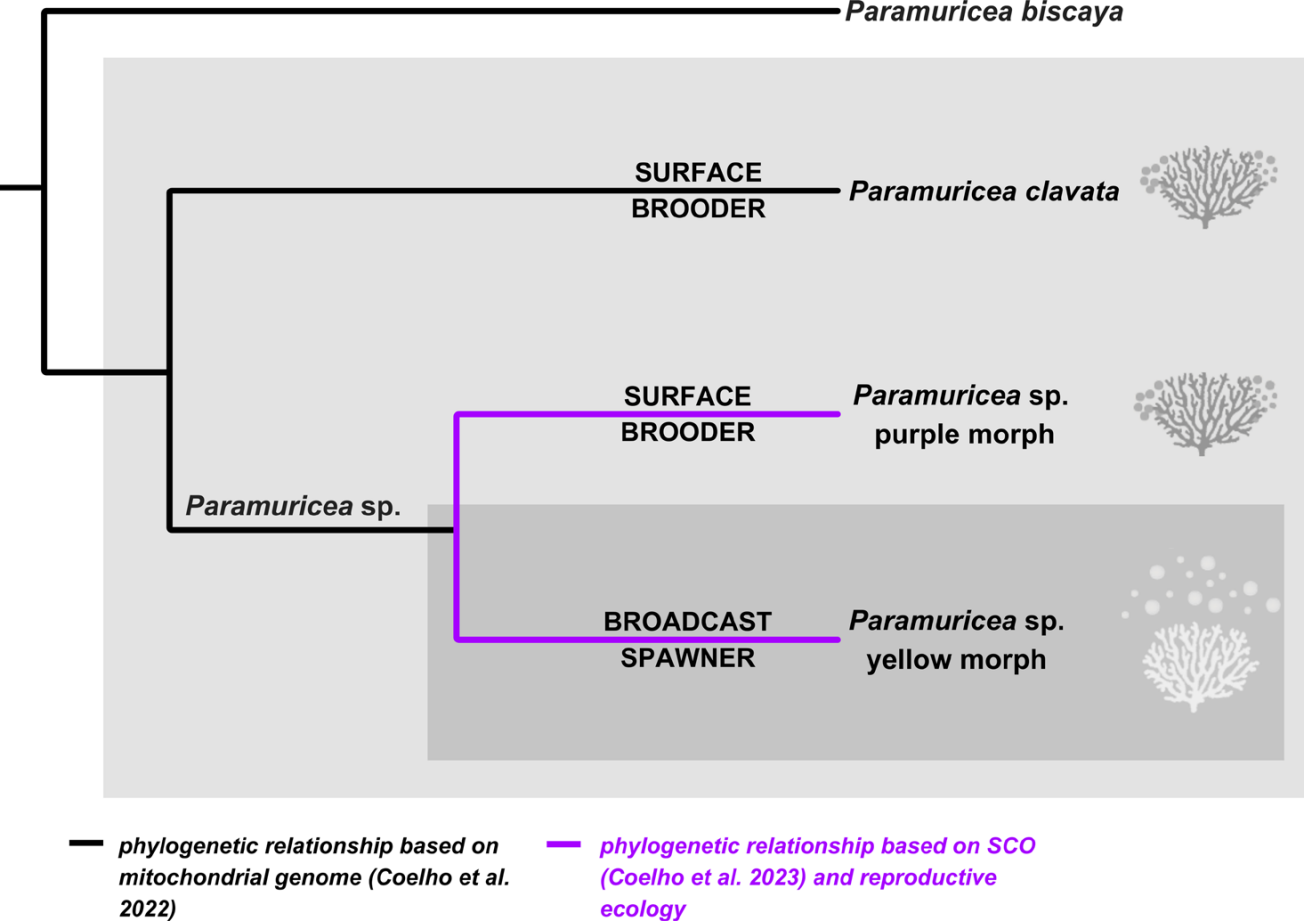
Phylogenetic relationship among closely related *Paramuricea* spp., redrawn from Coelho et al. (2022, 2023), with the reproductive modes superimposed on the tree. Reproductive modes are displayed for *P. clavata* from the Mediterranean and eastern Atlantic *Paramuricea* species.

**FIGURE 13:**
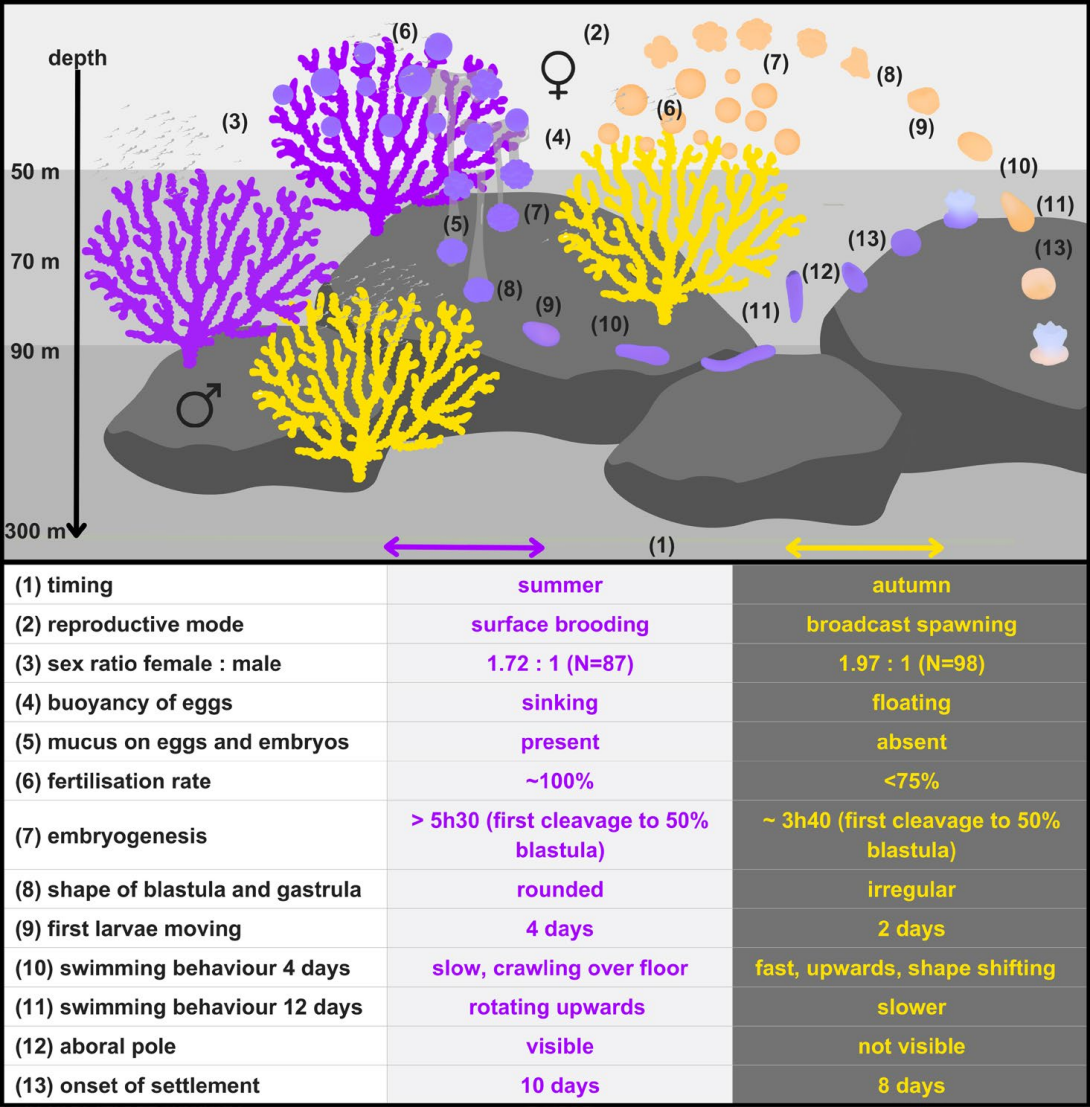
Summary of reproductive and early life differences between purple and yellow morphs of *Paramuricea* sp.

### 4.2 Differences in Adult Life‐History Traits

#### 4.2.1 Reproductive Timing

Our results show temporal divergence in reproductive periods between the two Atlantic *Paramuricea* morphs, suggesting this mechanism drives reproductive isolation between them. Over three consecutive years and for samples collected from the same overall geographic area (Figure 2), the purple morph (surface brooder) consistently spawned earlier than the yellow morph (broadcast spawner), with annual differences ranging from 17 to 41 days. Such a consistent temporal offset functions as a robust prezygotic barrier (Fukami et al. 2003; Gilmour et al. 2016; Rosser 2015) and represents one of the few mechanisms enabling sympatric coexistence while effectively preventing gene flow, a process that promotes long‐term reproductive and genetic divergence (Coyne and Orr 1998). Even minor shifts in spawning time, sometimes of only a few hours, can prevent fertilization in sympatric broadcast spawners, as gametes released by the first spawners can disperse before those of the later spawners are released (Levitan et al. 2004; Mercier and Hamel 2010). While temporal barriers between otherwise compatible species can occasionally become permeable under favorable environmental conditions (Fogarty, Lowenberg, et al. 2012; Fogarty, Vollmer, and Levitan 2012; Gilmour et al. 2016), in our study the morph‐specific reproductive window remained asynchronous over multiple years despite some degree of plasticity in the timing of reproduction. In 2023 and 2024, both morphotypes advanced their reproductive timing by about 1 month with respect to 2022, likely as a response to environmental cues such as temperature or food availability (Mercier and Hamel 2010; Sorek and Levy 2014). Alternatively, perhaps we did not capture the entire release period in 2022. This persistence of morph‐specific differences indicates that temporal isolation functions as a stable, and repeatable reproductive barrier.

#### 4.2.2 Fertilization Mode

In addition to temporal segregation, different reproductive modes can serve as isolating mechanisms reinforcing reproductive isolation between the two Atlantic *Paramuricea* morphotypes. Individuals of the purple morph displayed surface‐brooding behavior, releasing mucus‐coated eggs over an extended period (2–4 weeks) that were brooded on the maternal colonies or rapidly sank to the bottom of the tanks when detached from the colonies. In our experimental trials following embryogenesis, nearly complete fertilization success was achieved, a pattern typical of brooding corals (Lasker 2006). In contrast, the yellow morph is a broadcast spawner, releasing buoyant, non‐mucus‐coated eggs over only a few days, with a lower fertilization success (< 75%) in our experiments. In externally fertilizing corals, sperm dilution and rapid gamete aging restrict fertilization to short temporal windows (< 2 h), favoring tight synchronization (Levitan and Young 1995; Oliver and Babcock 1992). Thus, lower fertilization success in broadcast spawners is expected, whereas surface‐brooding concentrates gametes locally, increases fertilization efficiency, and reduces hydrodynamic loss. Both morphs exhibited male‐biased sex ratios (1.72:1 male to females in the purple morph and 1.92:1 in the yellow morph), consistent with other octocorals (Kahng et al. 2011), potentially mitigating sperm limitation (Rapuano et al. 2017).

### 4.3 Differences in Early Life‐History Traits

Embryos of the purple morph of *Paramuricea* developed more slowly, exhibited characteristic cleavage patterns, and produced elongated larvae with a pronounced apical pole. These larvae began slow crawling behavior from day 4 onward and showed only occasional upward rotations as well as a generally reduced upward movement behavior strongly reminiscent of the Mediterranean sister species *Paramuricea clavata* (Linares et al. 2008). Purple larvae settled later (~day 10) and had a shorter competence window (though this requires additional statistical validation). In contrast, embryos of the yellow morphotype developed more rapidly and exhibited distinct cleavage patterns. They produced compact, highly motile larvae that began active swimming as early as day 2 and settled between days 6–8, with a settlement‐competence period of up to 22 days. Interestingly, in the yellow morph, it appeared that the reduction in upward movement correlated with the onset of settlement competence (Figures 10 and 11).

These contrasting larval traits suggest fundamentally different dispersal strategies. Since coral larvae regulate vertical position through photo‐ and geotaxis, the observed differences in morphology and behavior provide a mechanistic basis for the distinct population structures typically observed in brooders versus broadcast spawners (Ayre and Hughes 2000; O'Donnell et al. 2025). The purple surface‐brooding morphotype, with its delayed motility, negative buoyancy, and benthic crawling behavior, exhibits a dispersal strategy that may promote larval philopatry similar to the sister species *P. clavata* , which is known for its fine‐scale genetic structuring and limited larval dispersal (Guizien et al. 2020; Linares et al. 2008; Mokhtar‐JamaÏ et al. 2011; Pérez‐Portela et al. 2016). By contrast, the yellow broadcast spawning morph, whose larvae develop and swim earlier, likely exhibits a broader dispersal radius and greater potential for long‐distance connectivity, as observed in other broadcast spawning relatives (Egger et al. 2025; MacLeod et al. 2024).

The pronounced differences observed in embryogenesis, larval morphology, and behavior show that divergence between the two morphs extends far beyond prezygotic incompatibility alone. These contrasts further illustrate how reproductive barriers can drive rapid divergence in early life‐history traits. In turn, such traits reinforce isolation, effectively minimize potential gene flow, and render secondary hybridization between the morphs highly unlikely (cf. Levitan et al. 2004). Taken together, our findings strongly suggest completed speciation that contributes to the maintenance of species separation. The precise mechanisms and drivers underlying such transitions remain poorly understood. Still, our results allow the formulation of testable hypotheses regarding the evolutionary factors that may have favored the shift from surface brooding to broadcast spawning and the evolution of divergent life‐history strategies.

### 4.4 Ecological Speciation—From Surface Brooder to Spawner

Broadcast spawning is generally regarded as the predominant ancestral reproductive mode of marine organisms, whereas brooding has evolved repeatedly and independently as a result of selection for fertilization assurance and local retention (Baird et al. 2009; Harrison and Wallace 1990; McHugh and Rouse 1998; Ostrovsky 2021). Reversals from brooding back to broadcast spawning are considered relatively rare, as brooding strategies involve profound ecological, physiological, and developmental adaptations that strongly constrain evolutionary returns to external fertilization (Strathmann 2015; Wray and Raff 1991).

In scleratinian corals, transitions from brooding to broadcast spawning are comparatively frequent, particularly in tropical regions of the Indo‐Pacific (Baird et al. 2009; Kerr et al. 2011). In contrast, in cold and deep Atlantic habitats, brooding (including surface brooding) dominates across many groups, especially among octocorals. This reproductive strategy has evolved multiple times independently in these environments and appears to confer adaptive advantages under such conditions (Baird et al. 2009; Cordes et al. 2001; Kahng et al. 2011; Roberts et al. 2006; Szmant 1986). Contrary to expectations, our results, together with the phylogenomic analyses of Coelho et al. (2023) and Quattrini et al. (2022) (Figure 12), indicate a transition from surface brooding to broadcast spawning in the yellow Atlantic *Paramuricea* morph. This pattern raises the hypothesis that strong selective pressures favoring dispersal and external fertilization have acted on this lineage. We further hypothesize that this transition may have been driven by the hydrodynamic conditions of southern Iberia, which are known to enhance the success of external fertilization (Levitan and Young 1995; Serrao et al. 1996).

The evolutionary shift in reproductive and early life‐history traits, together with the high genetic similarity, between the morphs, suggests an underlying genetic linkage but also argues against a prolonged period of geographic isolation during which reproductive isolation could have arisen as a by‐product of allopatric speciation (Schluter 2001; Wu 2001).

Ecological speciation over short spatial scales, driven by depth‐ or habitat‐related separation (parapatric) or by niche differentiation within the same habitat (sympatric), appears more likely (Dobzhansky 1982; Quattrini et al. 2013). Both mechanisms can enhance reproductive isolation upon secondary contact through positive selection on prezygotic barriers such as temporal divergence in reproduction and gametic incompatibility (Mercier and Hamel 2010; Schluter 2001). Environmental heterogeneity promotes local adaptation, which may restrict gene flow through habitat‐dependent parental effects (Prada and Hellberg 2013; Quattrini et al. 2013; Shlesinger and Loya 2021; Wilson and Hessler 1987). Fine‐scale environmental variation and depth gradients, combined with differing life‐history strategies, have repeatedly driven morph divergence and maintained biodiversity in deep‐sea corals, including congeneric species (Doughty et al. 2014; Pérez‐Portela et al. 2016; Quattrini et al. 2013, 2022; Radice et al. 2016).

Along the narrow Portuguese continental margin, several environmental gradients coincide with the distribution of the two *Paramuricea* morphotypes. The transition zone around Cabo de São Vicente is characterized by cold, upwelling‐dominated waters on the west coast and warmer, stratified conditions in the south (Relvas and Barton 2002, 2005; Relvas et al. 2007). With the complex seafloor topography, a narrow shelf with rocky ridges, small mounds, and sandy patches (Dias et al. 2020; Gonçalves et al. 2021; Monteiro et al. 2015), and rapidly shifting hydrographic conditions along the continental margin, this creates pronounced ecological contrasts.

The contrasting reproductive strategies of the two morphs provide different advantages and trade‐offs within this environmental setting. Surface brooding in the purple morph prolongs the reproductive period and increases offspring survival but is energetically costly and results in limited dispersal and stronger local genetic structuring (Coma et al. 1995; Mokhtar‐JamaÏ et al. 2011; Strathmann 2020). This may promote inbreeding and constrain adaptive potential under changing environmental conditions (Shlesinger and Loya 2021). In contrast, broadcast spawning like in the yellow morph increases dispersal and genetic exchange, enhancing connectivity, reproductive success, and resilience to disturbance (Lukoschek et al. 2016; Nakajima et al. 2016; Pinzón and LaJeunesse 2010). These traits facilitate recolonization and reduce extinction risk (O'Donnell et al. 2025; Pollock et al. 2017).

The most important oceanographic factor shaping reproductive strategies is water movement, which can rapidly dilute gametes and thereby reduce fertilization success (Oliver and Babcock 1992; Serrao et al. 1996). The extreme wave exposure of the shallow continental shelf along the west coast stands in strong contrast to the more sheltered conditions of the southern Portuguese coast and to the deeper water layers of the continental slope, where stable currents enable long‐distance larval transport (Levin 2006). This pronounced hydrodynamic gradient is likely a key selective force influencing the balance between broadcast spawning and brooding. Under highly energetic conditions, rapid gamete dispersal and loss can severely constrain reproductive success, favoring brooding strategies. In exposed reef habitats, proximity to parent colonies provides protection from strong currents, and even isolated brooding populations can maintain limited but meaningful gene flow through occasional larval exchange (McFadden et al. 2001; Oppen et al. 2008; Szmant 1986).

Local adaptation of the surface‐brooding lineage to specific microhabitats or depth zones may have initiated divergent selection and strengthened prezygotic barriers such as temporal isolation. In comparatively more sheltered and stable environments, reduced water movement may enhance fertilization efficiency and favor broadcast spawning. Improved dispersal and active larval swimming, enabling larvae to cross stratified water masses, likely contributed to the current distribution of the yellow morphotype in both shallow and deep habitats along the south and west coasts. Its broad depth range and probably large‐scale connectivity indicate a more generalist ecological strategy, whereas the purple morph remains more tightly associated with parental microhabitats.

Elucidating the precise mechanisms of habitat differentiation will require targeted in vivo ecological experiments that consider the specific microhabitats in which colonies establish.

## 5 Conclusion

Our comparative data show that differences in adult and larval life‐history traits generates strong reproductive isolation between a brooding (purple morph) and broadcast spawning (yellow morph) lineage. The presence of these reproductive barriers suggests that the two Atlantic *Paramuricea* morphs should be regarded as different species, with different evolutionary trajectories.

The shift in reproductive mode was accompanied by pronounced divergences in multiple early life‐history traits, including embryogenesis, larval behavior, and settlement, suggesting that many of these traits are likely genetically and functionally coupled. As such, this system provides a powerful empirical example of how selection acting on reproductive strategy can cascade across life‐history stages to promote lineage divergence. The two morphs may therefore represent a valuable model system for studying such genetic and ecological mechanisms linking reproductive modes, dispersal, and speciation, an area in which empirical data remain limited for anthozoans and other modular marine taxa (Nosil and Feder 2012).

## Author Contributions

**Christina Egger:** conceptualization (lead), data curation (lead), formal analysis (lead), investigation (lead), methodology (lead), supervision (lead), writing – original draft (lead), writing – review and editing (lead). **Aschwin H. Engelen:** conceptualization (supporting), funding acquisition (supporting), investigation (supporting), methodology (supporting), project administration (supporting), resources (supporting), supervision (equal), writing – review and editing (supporting). **Roland R. Melzer:** conceptualization (supporting), data curation (supporting), methodology (supporting), resources (supporting), supervision (supporting), visualization (supporting), writing – review and editing (supporting). **Marcellina Rola:** data curation (supporting), investigation (supporting). **Catarina Melo:** data curation (supporting), formal analysis (supporting), investigation (supporting). **Chiara Favaretto:** data curation (supporting), investigation (supporting), methodology (supporting). **Manuela Quiroga‐Pérez:** data curation (supporting), investigation (supporting). **Sheena Suet‐Wah Chung:** data curation (supporting), investigation (supporting). **Lorenzo Bramanti:** conceptualization (supporting), supervision (supporting), writing – original draft (supporting), writing – review and editing (supporting). **Ester A. Serrão:** funding acquisition (lead), project administration (lead), writing – original draft (supporting), writing – review and editing (supporting). **Márcio A. G. Coelho:** conceptualization (supporting), data curation (supporting), funding acquisition (supporting), investigation (supporting), methodology (supporting), project administration (supporting), resources (lead), supervision (equal), writing – original draft (supporting), writing – review and editing (supporting).

## Funding

Open access funding provided by FCT|FCCN (b‐on). This study received Portuguese national funds from FCT—Foundation for Science and Technology through contracts UID/04326/2025, UID/PRR/04326/2025, and LA/P/0101/2020 (DOI:10.54499/LA/P/0101/2020), Doctoral Scholarship SFRH/BD/151455/2021 to CE, CEECINST/0014/2018 to AHE as well as from funds from RESTORESEAS 2020–2021 Biodiversa+ and Water JPI joint call for research projects under the Bio‐divRestore ERA‐NET Cofund (grant agreement no. 101003777) and FCT (DivRestore/0013/2020); and CORALFORESTS (Fundação Belmiro de Azevedo).

## Conflicts of Interest

The authors declare no conflicts of interest.

## Figures and Tables

**FIGURE A1:**
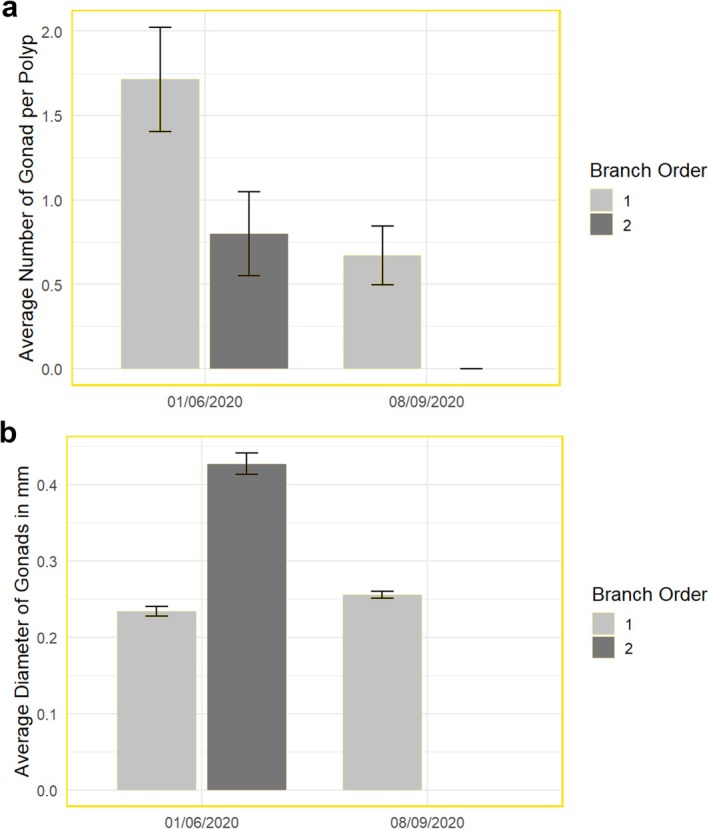
Spawning window of the yellow ± morph of *Paramuricea* sp. from near Cape Vincent, Sagres, Portugal in 2020. Polyp dissections of 3 colonies sampled on the 1st of June and 6 colonies from the 8th of September 2020 showing the decrease in average number (± SE) of oocytes per polyp (a) and average size (± SE) of oocytes (b) in first order (yellow color) and second‐order branches (orange color).

**FIGURE A2:**
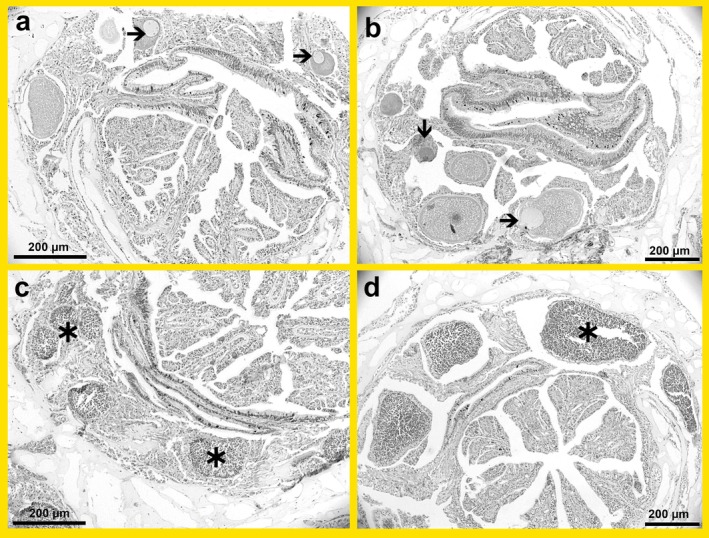
Spawning window of the yellow morphotype of *Paramuricea* sp. from near Cape Vincent, Sagres, Portugal in 2021. Histological sections of female and male polyps of specimens preserved on August 5, 2021 (a, c), and September 16, 2021 (b, f). Female polyps do show Stage 4 late vitellogenic oocyte (arrows) in early August (a), and mid‐September (b), and male polyps have visible spermatocytes (asterisk), but still small and growing (stage 2) in early August (c), and stage 3 mature spermatocytes in mid‐September (d).

## Data Availability

The data and code that support the findings of this study are openly available in Figshare at https://doi.org/10.6084/m9.figshare.30801329. The repository contains all datasets necessary to reproduce the results reported in this manuscript. Access to all files has been provided to editors and reviewers for the purposes of peer review. Relevant data are also included as Supporting Information.

## Appendix 1

### Inference of Reproductive Timing

#### Methods

We inferred the timing of reproduction in the yellow morph of *Paramuricea* sp. by examining gamete development stages in samples collected as fishing bycatch around Cape St. Vincent, southern Portugal, in 2020 and 2021 (see Dias et al. 2020 for details on fishing areas) at the Centro de Ciências do Mar do Algarve (CCMAR). Sampling targeted two time points, early summer and autumn, based on existing knowledge of reproductive patterns in Mediterranean gorgonian species (Gori et al. 2012; Ribes et al. 2007; Viladrich et al. 2022). We assessed gamete presence and maturation state through polyp dissection and histological sectioning at CCMAR. All samples were fixed in 10% neutral buffered formalin (Sigma‐Aldrich), washed three times with distilled water, and gradually dehydrated to 70% ethanol for further analysis. Polyps were dissected under a stereomicroscope to distinguish female and male individuals by identifying oocytes and spermaries, where possible. We then counted the number of gonads per polyp and determined branch order (*sensu* Brazeau and Lasker 1988). Individuals with high numbers of reproductive polyps were selected for histological sectioning. For histological analysis, branchlets approximately 2 cm long were cut from first‐ and second‐order branches of four colonies. Selected samples were hydrated and treated in 2.1 M EDTA (pH 8) for 2 days to decalcify sclerites embedded in the coral tissue, following the method used for sea bream scales (Vieira et al. 2011). After decalcification, samples were washed several times with deionized water to remove residual EDTA and then dehydrated through a graded ethanol series. Samples were saturated in xylene, impregnated, and embedded in low‐melting‐point paraffin wax (Histosec, Merck) using an automatic embedding processor. Serial sections of 5 μm were cut from the paraffin block using a manual rotary microtome (Leica RM 2135, Germany) and mounted on glass slides coated with 3‐aminopropyltriethoxysilane (APES; Sigma‐Aldrich, Madrid, Spain). For each wax block containing a coral branch, 2–3 sections were cut from the base of the polyps to sample gonadal tissue. Sections were dried overnight at 37°C, then cooled to room temperature for storage or staining. Slides were dewaxed and rehydrated before staining with hematoxylin and eosin (H&E) according to Najafpour et al. (2020), mounted in Tissue‐Tek Resin (Sakura Finetek), and covered with a glass coverslip. We examined the stained slides under a light microscope (Zeiss Observer D1) to identify reproductive structures of the polyps. The gametogenic maturation classification system defined by Waller (2005) was used to describe the gamete ripening process.

#### Results

Polyp dissections of samples of *Paramuricea* sp. yellow morphotype suggests that reproduction in 2020 occurred in early autumn as number of oocytes from early June to mid‐September showed a marked decrease irrespective of branch order: From 1.7 ± 0.3 average number (± SE) of female gametes per dissected polyp (*N* = 63) on 1st of June 2020 to 0.7 ± 0.2 (*N* = 115) on 8th of September 2020 in first order branches and from 0.8 ± 0.3 (*N* = 10) to 0 ± 0 (*N* = 59) in second‐order branches (Figure A1). The average diameter of oocytes in first branch order increased slightly from 0.23 mm ± 0.01 (average ± SE) in early June to 0.26 mm ± 0.01 in mid‐September. In second branches, gonad diameter was much larger compared with first order branches (0.43 mm ± 0.01) in early June but could not be compared with mid‐September because no more oocytes were found (Ab).

Histological sectioning of samples of *Paramuricea* sp. yellow morphotype from 2021 pointed towards a spawning occurring in early autumn or later, as female colonies are showing late stage 4 vitellogenic oocytes already on 5th of August and still on 16th of September (Figure A2c,d). The spermatocytes showed a ripening process from small, growing (stage 2) spermatocytes in early August until stage 3 spermatocytes in mid‐September (Figure A2e,f). Accelerated ripening may indicate the approach of the spawning period. In all the polyp dissections and histological sections made, we never observed embryos or larvae inside the polyps, which indicated that specimens of *P*. sp. yellow morph are not internal brooders.
